# Supplementary figures and images for: Sonographic Diagnosis and Follow-Up of a Rare Large Pre-Patellar Morel-Lavallée Lesion
Source: Diagnostics (Basel). 2025 Apr 1;15(7):883. doi: 10.3390/diagnostics15070883 (PMC11988603; doi:10.3390/diagnostics15070883)

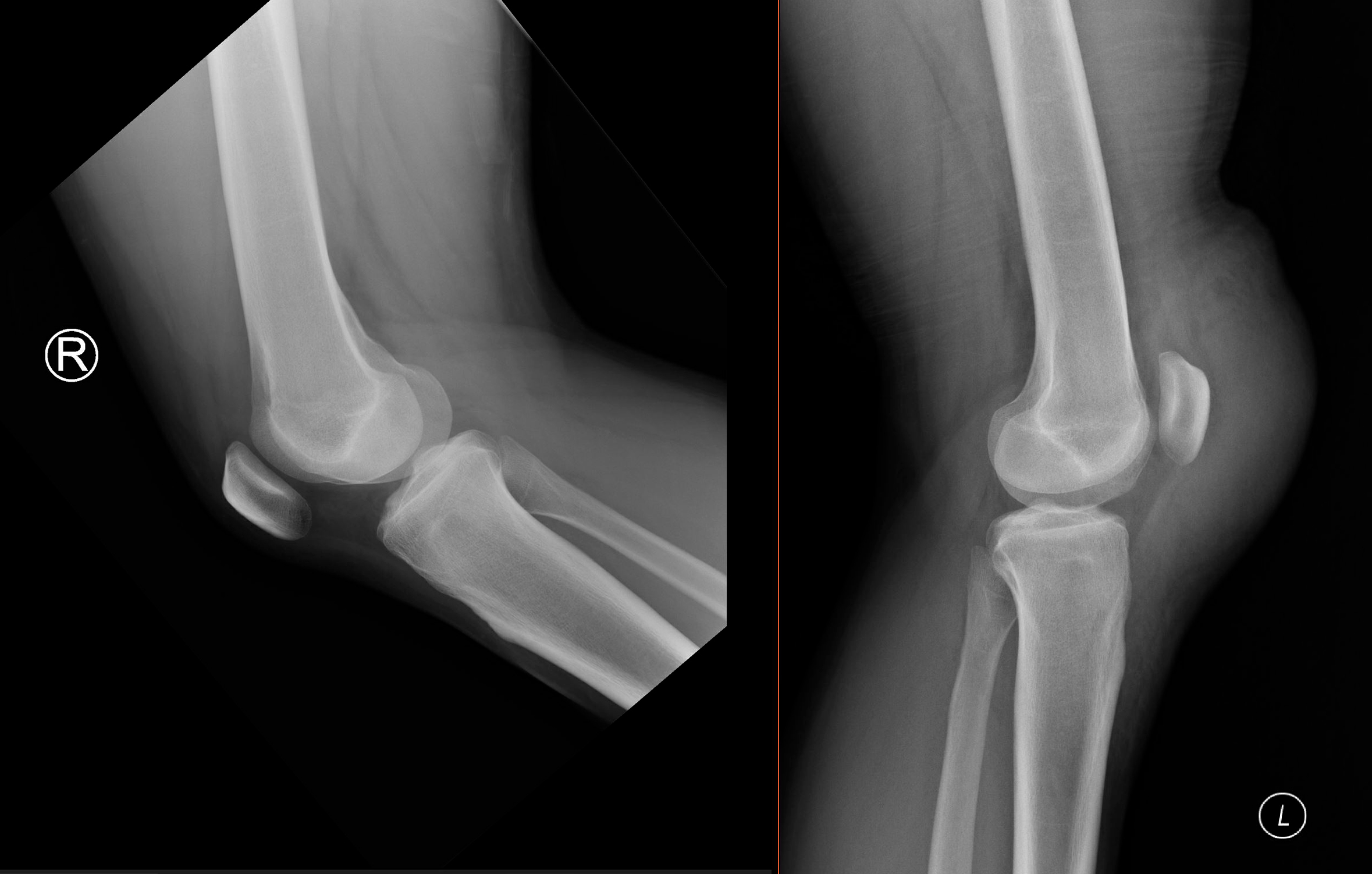

Supplement: Supplementary file 1 [file diagnostics-15-00883-s001.zip › Supplemental Material/Fig. 1S.jpg]
